# Cancer care: incidence, hospitalization and medical rehabilitation statistics for Germany from 2003 to 2016

**DOI:** 10.1186/s12913-026-15819-x

**Published:** 2026-10-14

**Authors:** Enno Nowossadeck, Benjamin Barnes

**Affiliations:** Department of Epidemiology and Health Monitoring, Robert Koch Institute, Nordufer 20, 13353 Berlin, Germany

**Keywords:** Cancer care, Incidence, Utilization, Inpatient care, Rehabilitation, Joinpoint regression

## Abstract

**Background:**

In Germany, inpatient hospital and rehabilitation facilities are two main settings for the delivery of cancer care. Epidemiology and demography are major forces that influence the need for care in these settings. This article examines the extent to which cancer incidence is associated with the use of inpatient and rehabilitative care.

**Methods:**

Population-based estimates of cancer incidence and survival came from the German Center for Cancer Registry Data. Statistical data on inpatient and rehabilitative care came from the Federal Statistical Office. Analyses included annual numbers and rates of hospitalizations and rehabilitation cases, and joinpoint regression models of these cases in relation to incident case numbers covering the period 2003–2016. To calculate the ratio of rehabilitation cases to incident cases, the number of incident cases was adjusted for 1-year survival.

**Results:**

The number of incident cancers increased from 2003 to 2016 (women: +9.6%; men: +7.0), whereas the difference in inpatient cases was far smaller (women −0.4%; men +4.5%). In 2016, there were 2.46 inpatient cases per incident case for women and 2.72 for men. This indicator varied: 1.87 for breast cancer (women only) and 3.33 for lung cancer (women and men). The estimated number of 1-year cancer survivors was 180,000 women and 185,900 men in 2016. The number of estimated cases in oncological rehabilitation per 100 1-year survivors among women increased from 59.2 (2003) to 65.2 (2010) and then decreased to 57.2 (2016). There was also an initial increase among men, from 50.8 (2003) to 51.5 (2008) and a subsequent decline to 46.9 (2016).

**Conclusions:**

Inpatient cases far exceed incident cases, which may reflect multiple hospitalizations for diagnostics and therapy of primary or recurring disease. The time trends of inpatient cases differ greatly from those of incident cases. This could be due to structural changes in the German health care system, such as possible shifts of services from the inpatient to the outpatient care sector. Regarding rehabilitation, the change in the trend of utilization has not been previously reported. Whether the ratio of rehabilitation cases to incidence indicates underuse of rehabilitative services cannot be assessed solely on the basis of the analyzed data.

**Supplementary Information:**

The online version contains supplementary material available at https://doi.org/10.1186/s12913-026-15819-x.

## Introduction

In 2016, approximately 492,000 people in Germany were newly diagnosed with cancer, and nearly 230,000 died from this disease [1]. The number of prevalent cases (10-year prevalence) was 1.444 million women and 1.437 million men (2016 [1]). Direct medical costs for malignant neoplasms amounted to €19.9 billion (2015 [2]). These statistics underscore the public health impact of cancer in Germany. As the number of incident cancer cases and cancer survivors increases, so too does the need for oncological care and rehabilitation [3]. However, the relationships among the number of incident cases and the utilization of inpatient oncological care and rehabilitation are poorly understood for Germany. These relationships can help to identify possible unmet care needs but must be used with caution when planning for future demand levels [4–6].

### Inpatient care

Inpatient care is one of the main pillars of health care in Germany [7]. With 1.28 million cases treated in 2016, malignant neoplasms are among the four most frequent diagnosis classes in inpatient care, along with cardiovascular diseases, diseases of the digestive system, and diseases of the musculoskeletal system and connective tissue [8]. Diagnosis and therapy of many cancers usually take place during at least one inpatient stay in a hospital in accordance with the respective treatment guidelines.

Inpatient oncology care in Germany has undergone structural changes in recent years, independent of epidemiological trends [9, 10]. Such changes in the structure of care include, for example, a shift of certain diagnostic and therapeutic services from the inpatient to the outpatient setting [11, 12], the establishment of Comprehensive Cancer Centers (CCC) that provide oncological care through a network of service providers [13] or the development of new diagnostic and therapeutic methods and procedures that make hospitalization unnecessary [9, 14]. In addition, the introduction of Diagnosis Related Groups (DRG) [15] has also provided economic incentives for hospitals to limit inpatient care to what is medically necessary [9, 16].

Temporal trends of case numbers in inpatient oncological care are therefore not only influenced by epidemiological trends, but also by structural changes in the provision of care. As a result, epidemiological and healthcare trends do not always run in parallel. Regarding projected demands for oncological care, analyses should therefore consider such variability in health care delivery [17].

### Rehabilitation

Persons with statutory health insurance in Germany who are newly diagnosed with cancer are entitled to medical rehabilitation [18], the aims of which are to positively influence quality of life, particularly regarding the physical, mental, social and occupational impacts of the disease or its therapy [19–22]. For persons of working age, rehabilitation also aims to maintain or improve the ability to work and reintegrate into working life [23].

Unlike in most other European countries, medical rehabilitation in Germany is predominantly provided in inpatient facilities [24]. Only about 12–14% of all rehabilitation patients are cared for in outpatient facilities [25, 26]. Another peculiarity of the rehabilitation system in Germany is that rehabilitation has to be applied for by the insured and cannot be prescribed by physicians [24]. After operations or serious illnesses treated in hospitals, health care staff (such as doctors and social services providers in the hospital) can inform patients about the possibility and goals of rehabilitation and encourage them to make use of it [24, 27].

### Aim

While previous studies have examined the utilization of rehabilitative care in oncology, most have focused on individual cancer types within a relatively small geographical area [28–37]. A comprehensive survey of rehabilitative care use at the population level, however, has not been conducted, and cancer registry data in Germany do not contain information on rehabilitative care. Although cancer registries do collect data on inpatient care, a nationwide dataset is still being compiled and does not yet provide a long time series. In this article, the available aggregated inpatient care and rehabilitation data sets and the epidemiologic data set are used together for the first time in an analysis with the goal of examining the utilization of inpatient care and rehabilitation in relation to cancer incidence in Germany. The aim of the article is to investigate the utilization of inpatient care and medical rehabilitation by cancer patients over time and in association with trends in cancer incidence.

## Methods

**Fig. 1:**
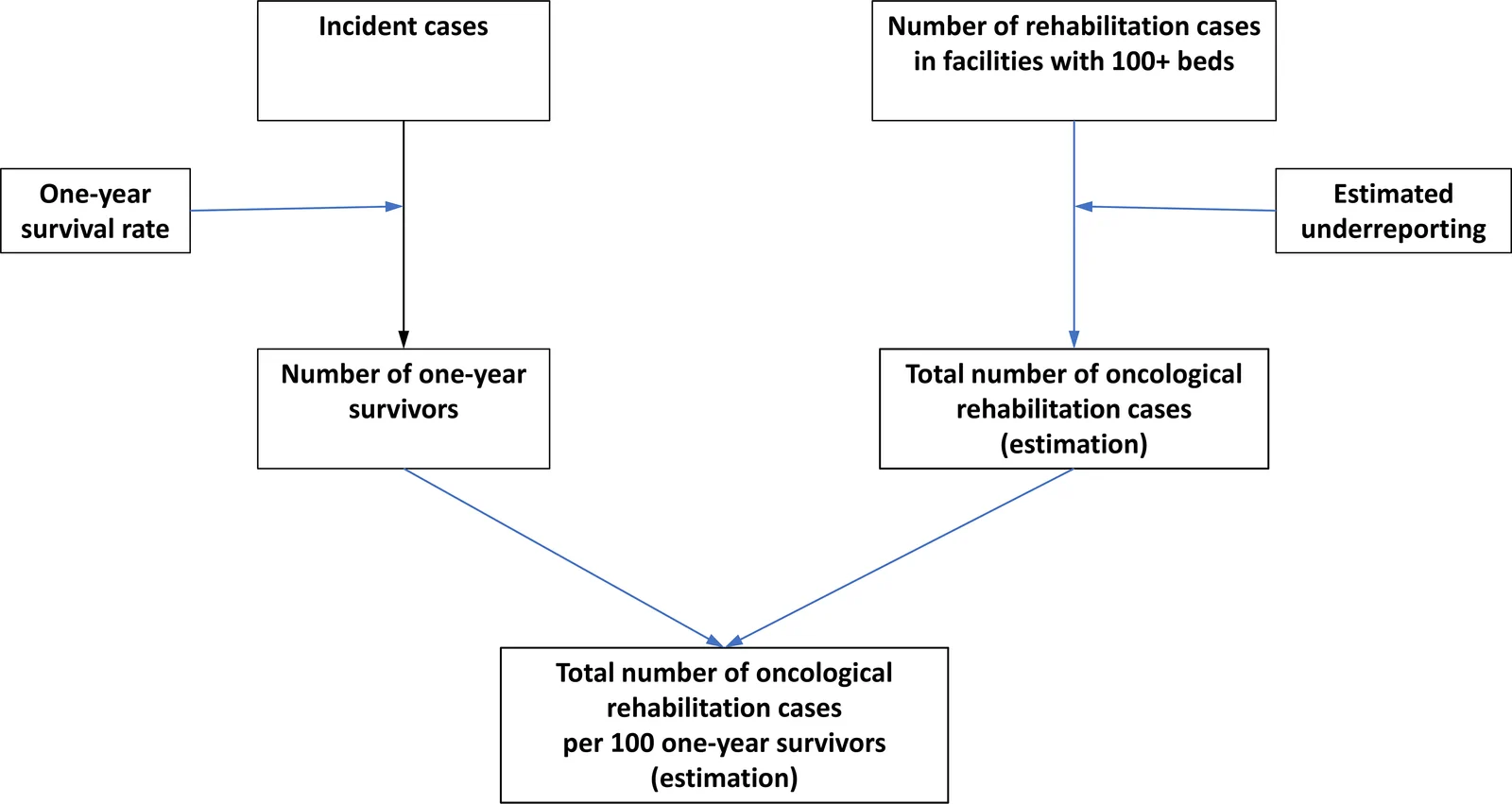
Methodology for estimating the ratio of the number of cancer rehabilitation cases to the number of 1-year survivors

To examine the use of inpatient and rehabilitative care in relation to cancer incidence, we merged aggregate data on treatment cases in the two care sectors with the number of incident cancer cases (Fig. 1). The merging was stratified by gender as well as by six age groups (under 45 years, 45–54, 55–64, 65–74, 75–84, and 85 years and older), by calendar year and by cancer diagnosis group. The following sections describe the data sources and analysis methods used.

### Data

The analyses include epidemiological data from the Centre for Cancer Registry Data at the Robert Koch Institute [38] and data on oncological care from the Federal Statistical Office of Germany (Destatis). The latter comprise inpatient “hospital diagnosis statistics” [8] and statistics on rehabilitation in facilities with more than 100 beds [39]. Inpatient care as an element of curative care and rehabilitation represent two separate sectors of health care in Germany. For this reason, the statistics are recorded separately from each other. These two statistics are case-based statistics, counting each time a patient receives inpatient or rehabilitative services. This means that multiple stays of the same patient are all included in the statistics, even if all services are provided to care for the same diagnosis. In contrast, the cancer registry data counts each cancer diagnosis only once. For the analysis of incidence and care, case numbers, crude rates and age-standardized rates based on the old European standard population were used.

Data were evaluated for all primary cancers except non-melanoma skin cancer (ICD-10 codes C00 - C97, excluding C44 and C77–C79) and for several individual cancer diagnoses: Colorectal (C18–C20), Lung (C33–C34), Breast (C50) and Prostate (C61). Supplementary Tables S1 and S2 contain results for additional diagnoses for reference.

#### Epidemiological data

Population-based estimates of cancer incidence and 1-year survival come from the Center for Cancer Registry Data at the Robert Koch Institute [38, 40]. These are publicly available in an online database (https://www.krebsdaten.de/Krebs/EN/). The data included here were published on 17 December 2019 [41]. German cancer registry data undergo quality checks to help detect underreporting, including reporting delays. The nationwide incidence data presented in this manuscript are estimates that incorporate corrections for incomplete registry data [40].

#### Inpatient care data

Data on inpatient care were drawn from the “hospital diagnosis statistics” of Destatis [8]. These statistics show cases of treatment broken down by gender, 5-year age group, calendar year and diagnosis. The diagnosis coded is the one that is mainly responsible for causing the inpatient hospital stay [8]. Inpatient hospital cases with a minimum length of stay of one day among residents of Germany were included. As an indicator for the utilization of inpatient care among cancer patients, the ratio of the number of hospital cases to the number of incident cases was calculated. Because cancer incidence is a person-based statistic and the “hospital diagnosis statistic” is a case-based statistic, the ratio between the number of hospital cases and the number of incident cases is expected to be greater than one. In the international literature, this ratio is often studied as a phenomenon of multiple inpatient stays (see review by Bell et al.2017 [42]) as an indicator of quality of care, especially from the perspective of unplanned multiple stays [43]. To our knowledge, there is a lack of information regarding multiple inpatient episodes in Germany and regarding the extent to which they can serve as an indicator of quality of care.

#### Rehabilitation data

Medical rehabilitation in Germany is provided by various institutions and in various forms, and there are no uniform and comprehensive statistics covering all forms of rehabilitation. Which institution is responsible for financing a particular rehabilitation case depends on the objective of the specific case. If the aim is to prevent a reduction in earning capacity, the German statutory pension insurance (Deutsche Rentenversicherung – DRV) is responsible. If, on the other hand, the aim is to prevent the need for long-term care and disability, the statutory health insurance (Gesetzliche Krankenversicherung – GKV) is usually responsible [24]. Of these two main providers of rehabilitation in Germany, only the DRV has published statistics with reference to diagnosis [24, 44], but these statistics do not cover all oncological rehabilitation cases. In the rehabilitation statistics published by the GKV there are no diagnosis-specific data for the analysis period.

In addition to statistical data from the above sources, diagnosis-specific data on rehabilitation cases are also available from the “hospital diagnosis statistics” of Destatis. These data report rehabilitation cases in facilities with more than 100 beds [39] and were used for this study. Although the rehabilitation dataset and the inpatient care dataset are both part of the “hospital diagnosis statistics”, these are separate datasets with data from different reporting institutions. For facilities with more than 100 beds there is a legal obligation to report rehabilitation statistics. For rehabilitation facilities with 100 or fewer beds as well as outpatient rehabilitation facilities, such a reporting obligation does not exist, and rehabilitation cases conducted in these settings are not included in the official “hospital diagnosis statistics” [44]. Using the methods outlined below, however, we have estimated the numbers of non-reported rehabilitation cases.

### Analysis methods

#### Estimation of rehabilitation figures

In order to estimate the number of rehabilitation cases in subpopulations missing from the hospital diagnosis statistics (rehabilitation in facilities with 100 or fewer beds and in outpatient facilities), data were taken from other publicly available rehabilitation statistics: rehabilitation cases in outpatient as well as in smaller inpatient facilities were obtained from the basic data of the hospital statistics of the Federal Statistical Office (“Grunddaten der Krankenhäuser”) [45] (no diagnosis-specific data); rehabilitation cases in outpatient facilities were obtained from the statistics of the German Pension Insurance [46], which is also available online [47], as well as data of the KG5 statistics of the Federal Ministry of Health [26] (cf [44]).

To estimate the total number of oncological rehabilitation cases in Germany, the number of rehabilitation cases in facilities with more than 100 beds is used as a starting point. Data for missing subpopulations are estimated by calculating available structural parameters for the (oncological) rehabilitation datasets and applying them to the missing subpopulations (Fig. 1, righthand side) [44].

For example, in 2016, 2% of all oncological rehabilitation cases took place in outpatient rehabilitation facilities [26, 47]. In the same year, the share of all rehabilitation cases that were conducted in facilities with 100 or fewer beds was 13% (corresponding to hospital statistics without reference to diagnosis [45]). These figures suggest that the rehabilitation statistics of Destatis (which only collects data from facilities with more than 100 beds) cover about 85% of all rehabilitation cases in Germany and thus an under-reporting of approximately 15% exists. Based on these assumptions, the total annual number of oncological rehabilitation cases in Germany was estimated.

This procedure for estimating the number of rehabilitation cases was applied in the same way for cancer as a whole and for the individual cancer diagnoses considered here (see Fig. 1, Table S3).

#### Calculation of the number of 1-year survivors

To justify participation in oncological rehabilitation, reintegration into working life or improvements in health impairment must be sufficiently possible [23]. In general, sufficient independence, mobility and capacity are prerequisites for oncological rehabilitation [48]. It has therefore been hypothesized here that cancer patients with a poor prognosis are less likely to apply for or utilize rehabilitation than those with a good or average prognosis. Therefore, we base our rehabilitation-related analyses on the estimated number of 1-year survivors rather than the total number of incident cases. To calculate the number of respective 1-year survivors, we calculated the 1-year survival rates with the individual cancer diagnoses according to the period approach and the methods in the report “Cancer in Germany” [1, 49]. For the calculations in this study, 1-year periods corresponding to calendar years 2003 to 2014 and a 2-year period covering the years 2015 and 2016 were defined. These survival probabilities were multiplied by the respective number of new cancer diagnoses to calculate the number of 1-year survivors. The estimated number of rehabilitation cases was then related to the number of 1-year survivors (see Fig. 1). Because of the introduction of the mammography screening program (see below for details) for women aged 50–69, separate analyses were performed for this age group as well as for adjacent age groups.

#### Outcome

The following ratios were calculated: a) number of inpatient treatment cases in hospitals relative to number of incident cases, b) number of estimated rehabilitation cases relative to 100 1-year survivors. Analyses were conducted for the period 2003 to 2016 because data are available for these years from all data sources used. No rehabilitation data are available prior to 2003, and 2016 was the latest available year of incidence estimates and survival estimates from the Center for Cancer Registry Data at the time of the data analyses. The two ratios (hereafter referred to as indicators of inpatient and rehabilitative care) were calculated annually for the years 2003 to 2016. These indicators were analysed as crude ratios without age standardization.

The three data bases used cover all residents of Germany. Therefore, the target population of the present analyses are all residents of Germany diagnosed with cancer.

#### Joinpoint regression models

Joinpoint regression models were used to identify possible trend changes in this time series [50]. This method can be used to identify not only time points at which an increasing trend changes to a decreasing one (or vice versa), but also time points at which the strength of the increase or decrease changes significantly. In addition, measures of change over time such as annual percent change (APC) or average annual percent change (AAPC) can be calculated.

#### Sensitivity analysis

To demonstrate the effect of including only 1-year survivors in the ratio of rehabilitation to incident cases, we conducted a sensitivity analysis including all incident cases. Rehabilitation-to-incidence ratios for colorectal, lung, breast and prostate cancers as well as for all cancers combined were re-calculated for the years 2003 through 2016.”

Calculations and analyses were performed using the National Cancer Institute’s Joinpoint Regression Program, version 4.6.0.0, and MS Excel 2019.

## Results

### All cancers combined

#### Incidence and 1-year survivors

**Fig. 2:**
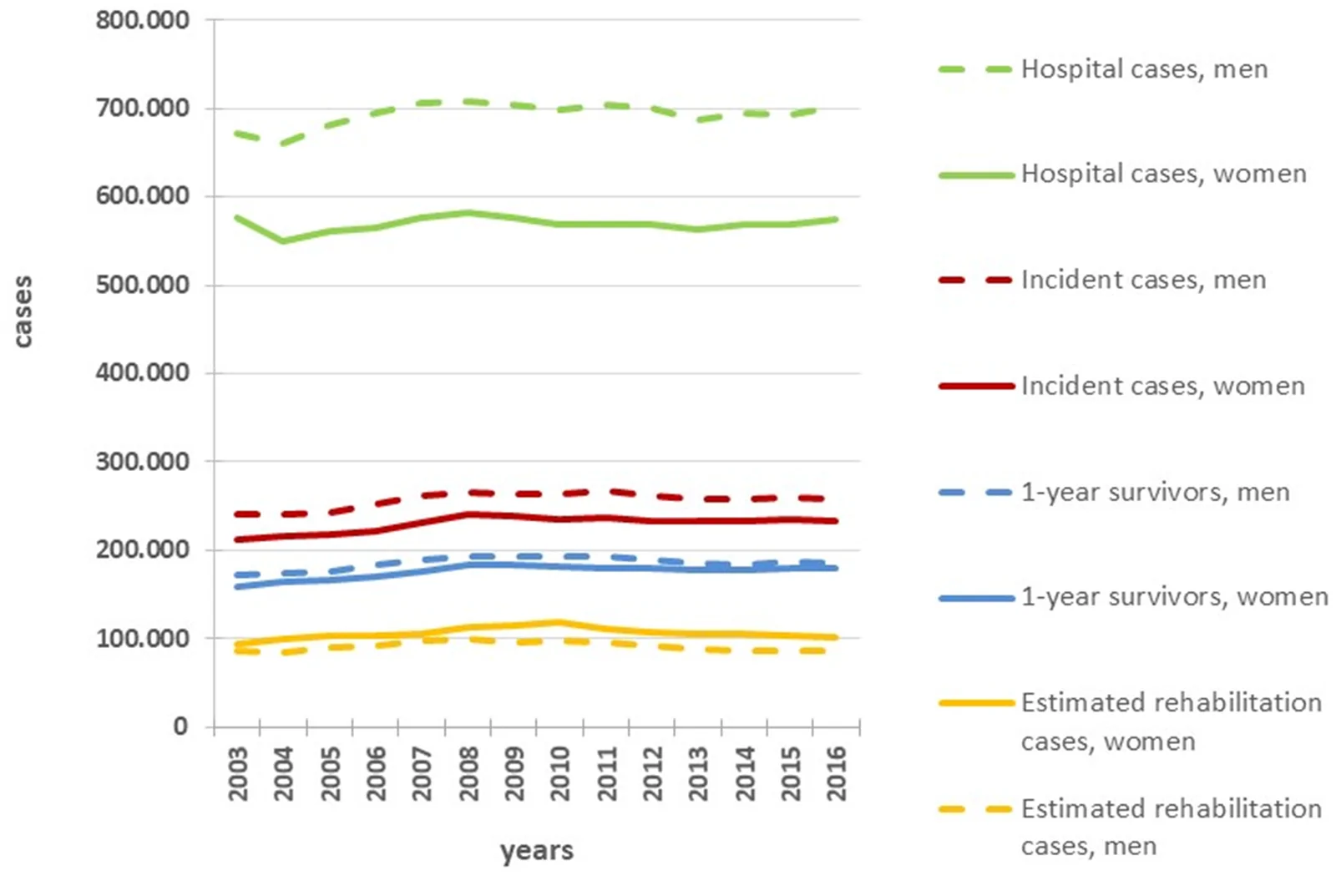
Trends in the number of incident cases, hospital cases, rehabilitation cases and 1-years survivors, 2003–2016, by sex

Trends in incidence figures for all primary cancers and for the individual cancers studied are shown in Tables 1 and S4 and Fig. 2. In the study period, 2003 to 2016, the age-standardized incidence rate for all primary cancers has remained fairly constant in women (+0.6%), and in men it has decreased by 13.7% (see Table S4). In contrast, the number of incident cases increased by 9.6% in women and by 7.0% in men during the same period (Table 1). Due to slight increases in average 1-year survival considering all cancers combined (C00–C97 excluding C44 and excluding C77–C79), the number of 1-year survivors increased to a larger extent than the number of incident cases: 12.0% among women and 8.5% among men.

**Table 1:** Epidemiological indicators and indicators of inpatient and rehabilitative care 2003 and 2016, for all cancers combined and selected diagnoses

Cancer	Woman		Man	
Indicator	2003	2016	2003	2016
**All cancers combined (C00–C97 excl. C44 and C77–C79)**				
Incident cases	213,073	233,572	241,720	258,524
1-year survivors	159,773	178,998	171,325	185,851
Inpatient hospital cases	576,151	574,109	672,348	702,815
Hospital cases per incident case	2.70	2.46	2.78	2.72
Rehabilitation cases (facilities with >100 beds)	80,459	87,539	74,023	73,481
Estimated total rehabilitation cases	94,600	102,463	87,018	87,158
Estimated rehabilitation cases per 100 1-year survivors	59.2	57.2	50.8	46.9
Estimated rehabilitation cases per 100 incident cases	44.4	43.9	36.0	33.7
**Colorectal cancer (C18–C20)**				
Incident cases	32,141	25,990	34,091	32,297
1-year survivors	25,295	21,263	26,263	25,282
Inpatient hospital cases	82,935	58,870	104,348	82,330
Hospital cases per incident case	2.58	2.27	3.06	2.55
Rehabilitation cases (facilities with >100 beds)	11,042	8,222	11,544	9,230
Estimated total rehabilitation cases	12,482	9,366	13,053	10,676
Estimated rehabilitation cases per 100 1-year survivors	49.3	44.0	49.7	42.2
Estimated rehabilitation cases per 100 incident cases	38.8	36.0	38.3	33.1
**Lung cancer (C33–C34)**				
Incident cases	13,064	21,497	34,429	35,962
1-year survivors	5,690	10,058	13,894	14,104
Inpatient hospital cases	42,292	71,640	112,323	119,800
Hospital cases per incident case	3.24	3.33	3.26	3.33
Rehabilitation cases (facilities with >100 beds)	2,264	4,497	4,913	6,094
Estimated total rehabilitation cases	2,662	5,264	5,777	7,228
Estimated rehabilitation cases per 100 1-year survivors	46.8	52.3	41.6	51.3
Estimated rehabilitation cases per 100 incident cases	20.4	24.5	16.8	20.1
**Breast cancer (C50) - Women / Prostate cancer (C61) - Men**				
Incident cases	61,760	68,948	64,966	58,776
1-year survivors	58,032	64,158	60,365	54,064
Inpatient hospital cases	145,834	129,167	83,428	74,559
Hospital cases per incident case	2.36	1.87	1.28	1.27
Rehabilitation cases (facilities with >100 beds)	38,795	43,656	26,417	25,118
Estimated total rehabilitation cases	45,617	51,099	31,064	29,793
Estimated rehabilitation cases per 100 1-year survivors	78.6	79.2	51.5	55.1
Estimated rehabilitation cases per 100 incident cases	73.9	74.1	47.8	50.7

Source: Own calculations; Data basis: German Center for Cancer Registry Data [38], hospital diagnosis statistics [8], diagnosis data of preventive or rehabilitation facilities with more than 100 beds [39]

#### Inpatient care

In 2016, 574.1 thousand inpatient cases due to cancer were recorded for women and 702.8 thousand for men (Table 1). Results for various cancer diagnoses are shown in Table S1.

**Fig. 3:**
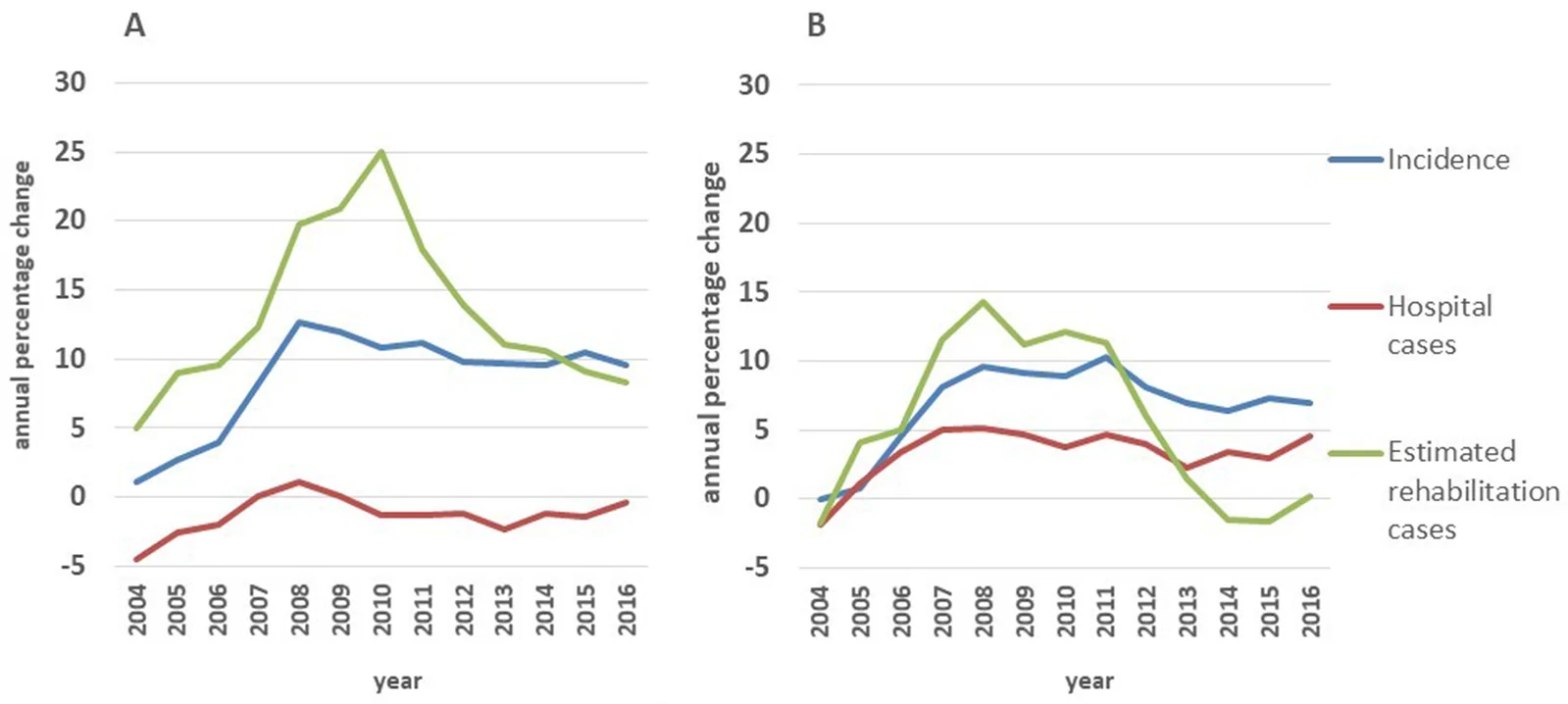
Annual percentage change from 2003 in the number of incident cases as well as inpatient and rehabilitation cases 2004–2016 (all cancers combined). A – Women; B – Men

**Fig. 4:**
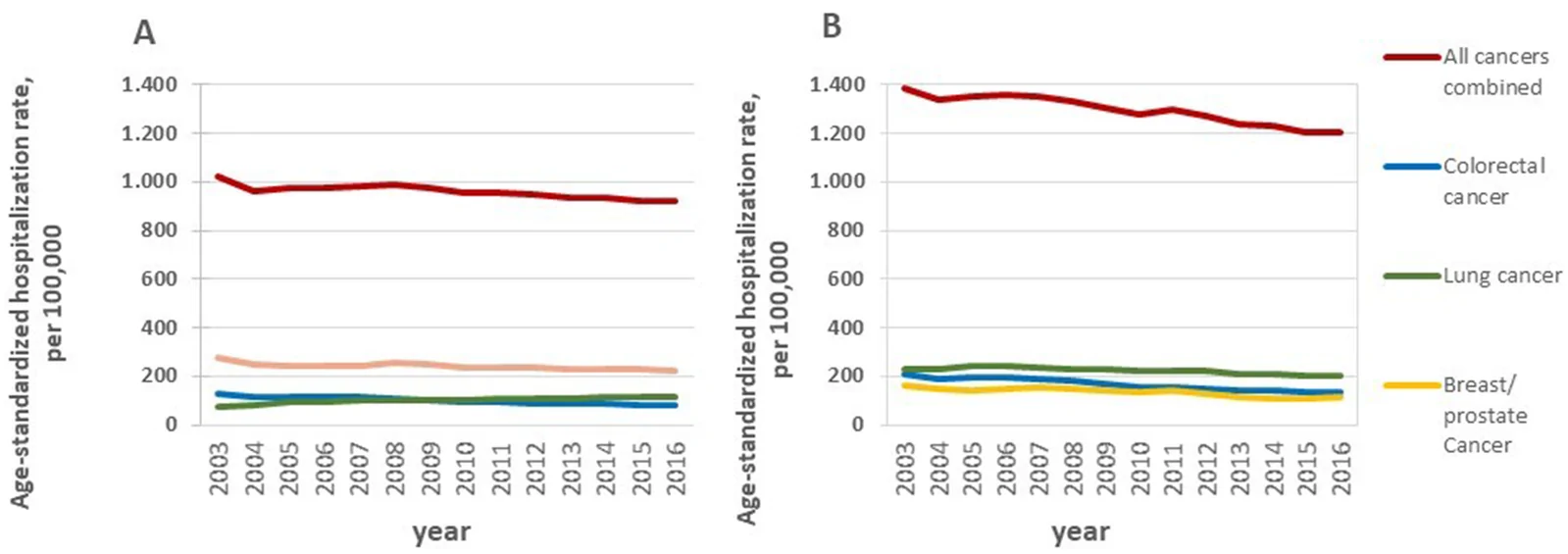
Age-standardized hospitalization rates per 100,000 persons for all cancers combined and for selected diagnoses, 2003–2016, A - Women; B - Men

Over the period 2003 to 2016, the number of inpatient cases due to cancer has changed little for both women (−0.4%) and men (+4.5%) (see Table 1, Figs. 2 and 3). Age-standardized cancer treatment rates declined, from 1,023 (women) and 1,386 (men) cases per 100,000 population in 2003 to 923 (women) and 1,205 (men) cases per 100,000 population in 2016 (Fig. 4), a decrease of 9.8% for women and 13.1% for men.

**Fig. 5:**
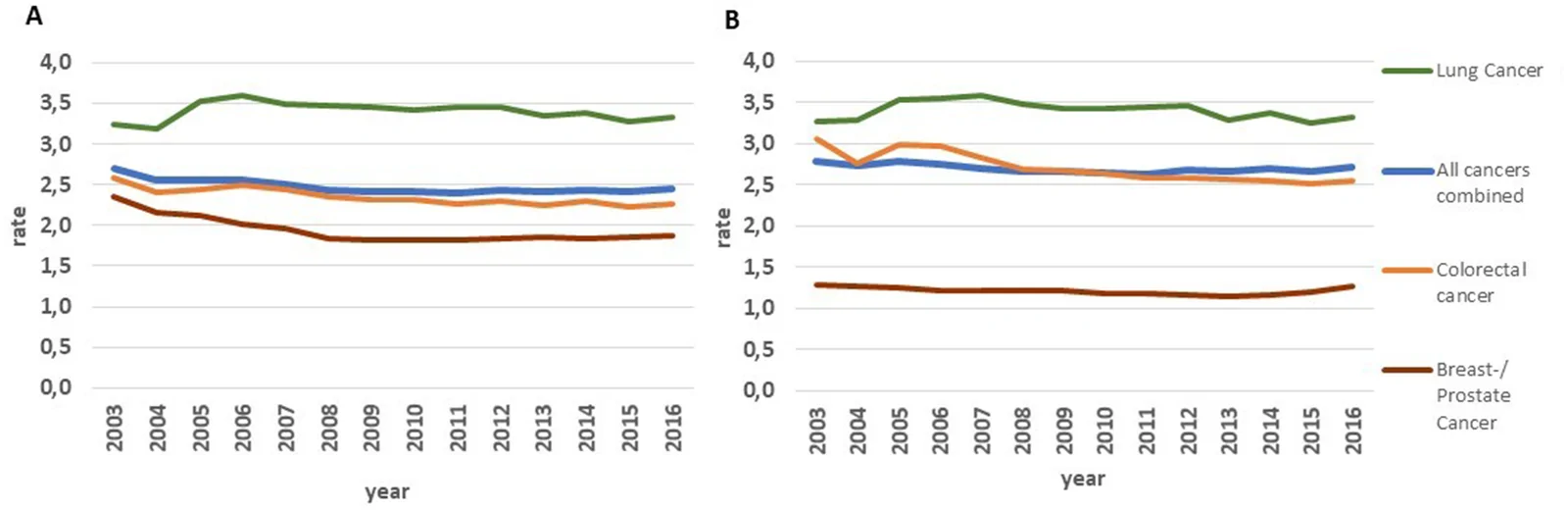
Number of hospital cases relative to the number of incident cases, 2003–2016, by types of cancer. A – Women; B – Men

In 2016, the number of inpatient cancer cases was 2.46 times higher than the number of incident cases in women and 2.72 times higher in men (Table 1, Fig. 5). The ratio between the number of inpatient cases and the number of incident cases differs considerably between cancer diagnoses (cf. Table 1). Results of the joinpoint analysis of inpatient care caseloads for the four diagnoses studied here are presented in Table 2.

**Table 2:** Results of joinpoint analysis of trends in incidence as well as inpatient and rehabilitation cases by sex and cancer diagnosis (ICD-10), 2003–2016

Indicator	Sex	ICD-10	Segment	Lower endpoint	Upper endpoint	APC	Lower CI	Upper CI	Test statistic (t)	Prob > |t|
Incident	Women	C18–C20	1	2003	2008	−0.9	−1.3	−0.4	−4.3	0.005
cases			2	2008	2012	−2.9	−4.0	−1.7	−5.9	0.001
			3	2012	2016	−1.2	−2.0	−0.5	−4.2	0.006
		C33–C34	1	2003	2007	**4.8**	3.3	6.2	7.6	<0.001
			2	2007	2016	**3.4**	3.1	3.8	21.9	<0.001
		C50	1	2003	2005	−0.2	−3.6	3.4	−0.1	0.894
			2	2005	2008	**7.3**	3.6	11.1	4.9	0.003
			3	2008	2016	**−1.2**	−1.5	−0.8	−8.6	0.001
	Men	C18–C20	1	2003	2008	**1.1**	0.4	1.8	3.6	0.006
			2	2008	2016	**−1.4**	−1.7	−1.1	−10.3	<0.001
		C33–C34	1	2003	2009	**0.8**	0.4	1.1	5.1	0.001
			2	2009	2016	0.1	−0.2	0.3	0.7	0.528
		C61	1	2003	2009	2.0	−0.4	4.4	1.9	0.093
			2	2009	2016	**−2.9**	−4.7	−1.1	−3.6	0.006
Inpatient	Women	C18–C20	1	2003	2007	−1.5	−3.0	0.1	−2.4	0.056
cases			2	2007	2012	**−3.9**	−5.3	−2.4	−6.3	0.001
			3	2012	2016	−1.3	−2.8	0.2	−2.1	0.084
		C33–C34	1	2003	2006	**9.0**	7.4	10.7	12.9	<0.001
			2	2006	2016	**2.8**	2.5	3.0	30.4	<0.001
		C50	1	2003	2005	**−4.9**	−7.3	−2.4	−6.3	0.008
			2	2005	2008	**2.9**	0.7	5.1	4.2	0.024
			3	2008	2011	−2.1	−4.1	0.1	−3.1	0.054
			4	2011	2016	**−0.5**	−0.8	−0.2	−5.0	0.015
	Men	C18–C20	1	2003	2007	0.6	−1.1	2.4	0.8	0.436
			2	2007	2011	**−3.8**	−6.6	−0.9	−3.2	0.018
			3	2011	2016	**−1.4**	−2.6	−0.2	−3.0	0.025
		C33–C34	1	2003	2006	**4.5**	3.5	5.6	9.7	<0.001
			2	2006	2016	**−0.6**	−0.7	−0.5	−12.2	<0.001
		C61	1	2003	2011	0.1	−1.4	1.7	0.2	0.844
			2	2011	2014	−7.0	−18.7	6.5	−1.3	0.238
			3	2014	2016	6.5	−6.9	22.0	1.1	0.296
Rehabilitation	Women	C18–C20	1	2003	2006	−0.2	−2.1	1.7	−0.2	0.817
cases			2	2006	2016	**−3.0**	−3.2	−2.8	−31.3	<0.001
		C33–C34	1	2003	2008	**7.1**	5.4	8.8	9.8	<0.001
			2	2008	2016	**4.4**	3.6	5.1	13.4	<0.001
		C50	1	2003	2010	**4.6**	3.7	5.6	11.8	<0.001
			2	2010	2013	−5.7	−12.2	1.2	−2.0	0.088
			3	2013	2016	−0.7	−4.1	2.9	−0.5	0.643
	Men	C18–C20	1	2003	2009	0.4	−0.3	1.1	1.3	0.213
			2	2009	2016	**−2.8**	−3.3	−2.3	−12.5	<0.001
		C33–C34	1	2003	2006	**4.8**	3.6	6.1	9.7	<0.001
			2	2006	2011	**2.1**	1.4	2.7	7.9	<0.001
			3	2011	2016	**−0.5**	−0.9	−0.0	−2.5	0.046
		C61	1	2003	2008	**5.8**	3.7	7.9	8.9	0.003
			2	2008	2011	−3.2	−12.4	7.1	−1.0	0.383
			3	2011	2014	−9.3	−18.0	0.3	−3.1	0.053
			4	2014	2016	3.6	−6.4	14.6	1.1	0.347

Bold: *p*-value < 0.05; APC: Annual percent change; ICD-10: International Statistical Classification of Diseases and Related Health Problems 10th Revision; CI: Confidence interval. Data basis: German Center for Cancer Registry Data [38], hospital diagnosis statistics [8], diagnosis data of preventive or rehabilitation facilities with more than 100 beds [39]

The ratio of the number of inpatient cases to the number of incident cases has fallen continuously over the period studied, more so for women than for men, with a slight increase in 2016 (Fig. 5). Whereas in 2003 the values of this ratio were 2.70 (women) and 2.78 (men), in 2016 they were 2.46 (women) and 2.72 (men). The results of the joinpoint analyses are shown in Supplementary Figure S1.

#### Rehabilitation

In facilities with more than 100 beds, 87.5 thousand rehabilitation cases relating to cancer were documented for women and 73.5 thousand for men in 2016 (Table 1). The results for different cancer diagnoses can be seen in Table S1. The results of the joinpoint analyses are shown in Supplementary Figure S5–S8.

The total number of oncological rehabilitation cases, including facilities with fewer than 100 beds and outpatient rehabilitation, was estimated to be 102.5 thousand for women and 87.2 for men in 2016 (Table S3). Estimates for 2003 and for men can be seen in Table 1. The estimated total number of rehabilitation measures for primary cancers per 100 1-year survivors in 2016 was 57 for women and 47 for men (see Table 1).

The ratio between rehabilitation cases and 1-year survivors differs between diagnoses, in some cases considerably. For example, in 2016, for breast cancer among women, there were 79 estimated rehabilitation cases per 100 1-year survivors (see Table 1 and Table S2). The ratios for colorectal cancer and lung cancer among women were 44 and 52, respectively. The figures for men for colorectal cancer (42) and lung cancer (51) are close to those for women.

**Fig. 6:**
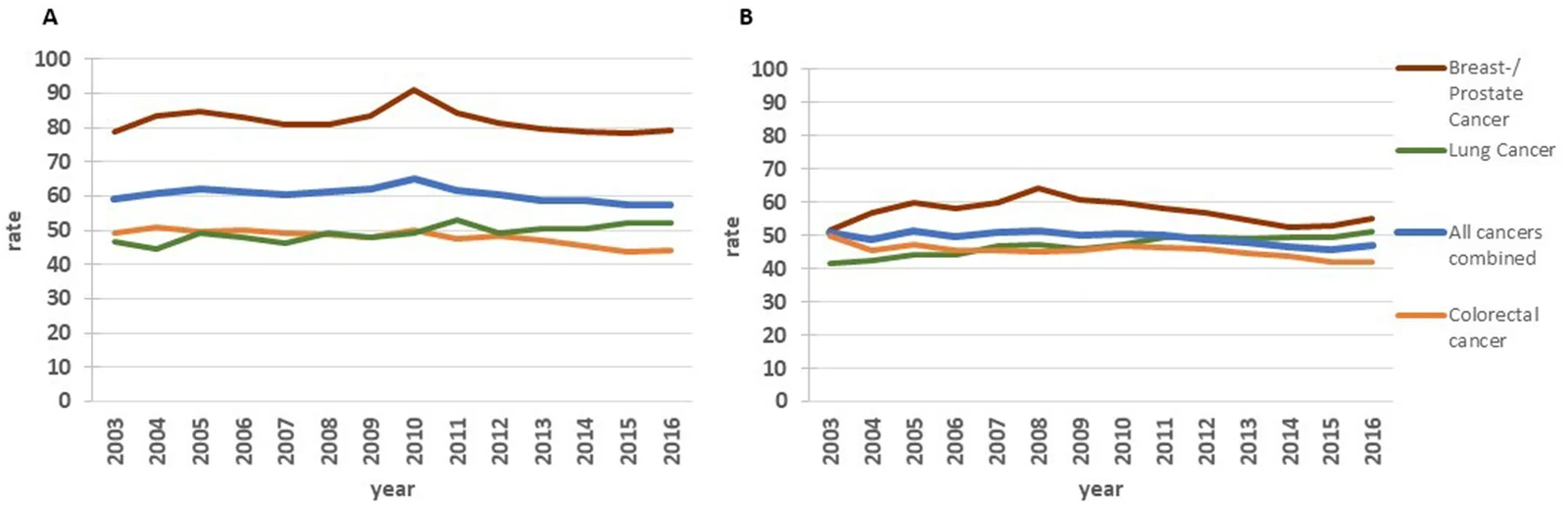
Estimated rehabilitation cases relative to 100 1-year survivors, 2003–2016, by types of cancer. A – Women; B – Men

The number of estimated cases in oncological rehabilitation among women increased until 2010 and then decreased (Fig. 2). This trend is reflected in the ratio of estimated rehabilitation cases to the number of 1-year survivors (Fig. 6). In the case of men, the values have fluctuated over the period studied, with a slight downward trend since 2007 (cf. Fig. 6). Results of joinpoint analyses of rehabilitation indicators for the four diagnoses studied here are provided in Table 3 and the Supplementary Figures S5–S8.

**Table 3:** Results of joinpoint analyses of trends in ratios of inpatient or rehabilitative care to incident cases by sex and cancer diagnosis (ICD-10), 2003–2016

Indicator	Sex	ICD-10	Segment	Lower endpoint	Upper endpoint	APC	Lower CI	Upper CI	Test statistic (t)	Prob > |t|
Hospital cases /	Women	C18–C20	1	2003	2011	**−1.3**	−1.8	−0.8	−5.7	<0.001
incident case			2	2011	2016	−0.2	−1.3	0.9	−0.5	0.662
		C33–C34	1	2003	2006	**3.9**	1.8	6.0	4.2	0.002
			2	2006	2016	**−0.8**	−1.0	−0.5	−7.5	<0.001
		C50	1	2003	2009	**−4.0**	−4.4	−3.5	−20.3	<0.001
			2	2009	2016	**0.5**	0.2	0.9	3.3	0.010
	Men	C18–C20	1	2003	2011	**−1.8**	−2.6	−1.0	−5.1	0.001
			2	2011	2016	−0.6	−2.4	1.2	−0.7	0.482
		C33–C34	1	2003	2006	**3.4**	1.3	5.6	3.7	0.005
			2	2006	2016	**−0.9**	−1.1	−0.7	−8.8	<0.001
		C61	1	2003	2014	**−1.0**	−1.2	−0.8	−11.4	<0.001
			2	2014	2016	**5.1**	1.3	9.1	3.0	0.014
Rehabilitation	Women	C18–C20	1	2003	2012	**−0.5**	−0.8	−0.3	−4.4	0.002
cases / 100			2	2012	2016	**−2.5**	−3.6	−1.4	−5.0	0.001
1-year survivors		C33–C34	1	2003	2005	1.9	−7.2	11.9	0.5	0.660
			2	2005	2016	**0.9**	0.5	1.3	5.4	<0.001
		C50	1	2003	2010	0.9	−0.2	1.9	1.9	0.093
			2	2010	2016	**−1.7**	−3.0	−0.4	−2.9	0.017
	Men	C18–C20	1	2003	2008	**−1.2**	−2.0	−0.5	−4.1	0.006
			2	2008	2011	1.4	−3.6	6.6	0.7	0.533
			3	2012	2016	**−2.4**	−3.1	−1.6	−7.9	<0.001
		C33–C34	1	2003	2007	**2.8**	1.0	4.6	3.5	0.006
			2	2007	2016	**1.0**	0.5	1.5	4.4	0.002
		C61	1	2003	2008	**3.1**	0.8	5.5	3.0	0.014
			2	2008	2016	**−2.3**	−3.3	−1.2	−4.7	0.001

Bold: *p*-value < 0.05; APC: Annual percent change; ICD-10: International Statistical Classification of Diseases and Related Health Problems 10th Revision; CI: Confidence interval. Data basis: German Center for Cancer Registry Data [38], hospital diagnosis statistics [8], diagnosis data of preventive or rehabilitation facilities with more than 100 beds [39]

### Colorectal cancer

#### Incidence and 1-year survivors

**Fig. 7:**
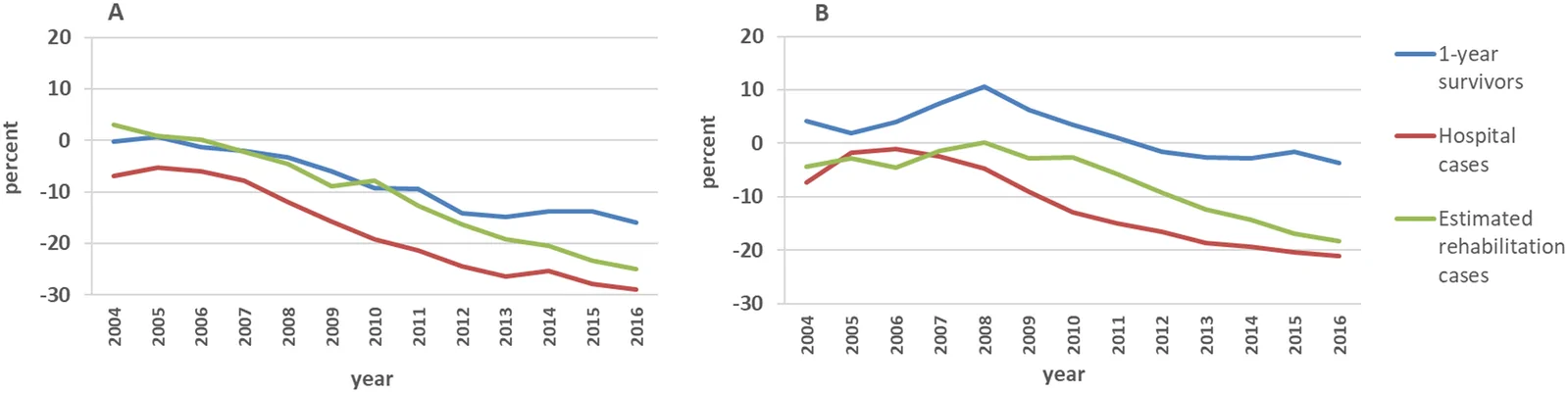
Annual percentage change from 2003 in the number of incident cases as well as inpatient and rehabilitation cases, colorectal cancer, 2004–2016. A – Women; B – Men

In 2016, 32,297 incident cases of colorectal cancer were diagnosed among men and 25,990 among women. Since 2003, the number of incident colorectal cancer cases steadily decreased among women, whereas among men there was an increase through 2008 followed by a decrease (Fig. 7, Supplementary Table S4). Joinpoint analysis showed a change in trend for both women and men in 2008, with an additional change among women in 2012. For the period through 2016, the observed decline was significant only among men (Table 2). Trends in the estimated number of 1-year survivors are similar due to fairly constant survival estimates.

#### Inpatient care

For hospital cases for colorectal cancer, joinpoint regression identified three periods with different trends for both women and men. For women, there was a significant decrease in the middle period (2007–2012: APC = −3.9%, 95%CI: −5.3 to −2.4%), and for men, the decrease was significant in the last two periods (2007–2011, APC = −3.8, 95%CI: −6.6 to −0.9) and 2011–2016: APC = −1.4, 95%CI: −2.6 to −0.2) (Table 2).

The ratio between the number of inpatient cases and the number of incident colorectal cancer cases in 2016 was 2.27 for women and 2.55 for men. This indicator shows a decreasing trend: The AAPC for the period 2003 to 2016 is −0.9% (95% confidence interval (CI): −1.3 to −0.4%) in women and −1.3% (95% CI: −2.1 to −0.6%) in men. Up to 2011, this trend was significant for both women and men, but not significant from 2011 onwards (see Table 3 and Supplementary Figures).

For colorectal cancer, the number of rehabilitation cases per 100 1-year survivors in 2016 was 44 among women and 42 among men (see Table 1 and Table S2). This ratio declined slightly over the analysis period for both women and men (Table 3, Fig. 6) (AAPC women: −1.2 (95%CI: −1.5 to −0.8); men −1.1 (95%CI: −1.1 to −0.1)).

### Lung cancer

#### Incidence and 1-year survivors

**Fig. 8:**
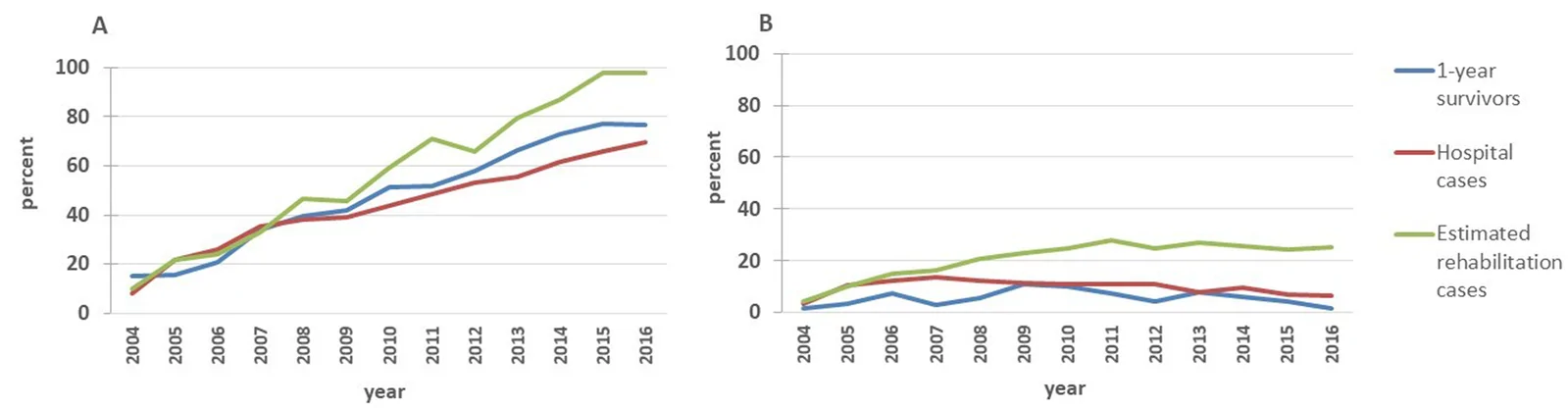
Annual percentage changes from 2003 in the number of incident cases as well as inpatient and rehabilitation cases, lung cancer, 2004–2016. A – Women; B – men

The trend in age-standardized incidence rates of lung cancer between 2003 and 2016 differs between women and men. There was an increase of 46.7% in women and a decrease of 16.7% in men (Supplementary Table S4). The number of incident cases, however, has increased over this period for both women and men. Joinpoint regression identified two periods with differing trends for both women (2003–2007, 2007–2016) and men (2003–2009, 2009–2016). For women, there is a significant increase in both periods; for men, there is an increase in both periods that is significant only in the first (Table 2). The number of 1-year survivors increased similarly to incident cases among women, but the trend in men was not as clear (Fig. 8).

#### Inpatient care

The ratio of inpatient hospital cases to the number of incident cases in 2016 was 3.33 for both women and men with lung cancer (Table 1). Joinpoint analysis of this indicator shows strongly increasing values through the year 2006 for women and men, followed by slightly decreasing values (cf. Table 3 and Supplementary Figures). Due to this trend reversal, there is no change for the total period (AAPC women: 0.3% (95% CI −0.1 to 0.7); men: −0.1% (95% CI −0.3 to 0.5)).

#### Rehabilitation

Among women the number of rehabilitation cases per 100 1-year lung cancer survivors was 52. The figure for men (51) is similar to that for women (see Table 1 and Table S2). This ratio increased slightly over the analysis period for both women and men (Fig. 6) (AAPC women: 1.0 (95%CI: −0.2 to 2.3; men: 1.5 (95%CI: 1.0 to 2.1)).

### Breast cancer

#### Incidence and 1-year survivors

**Fig. 9:**
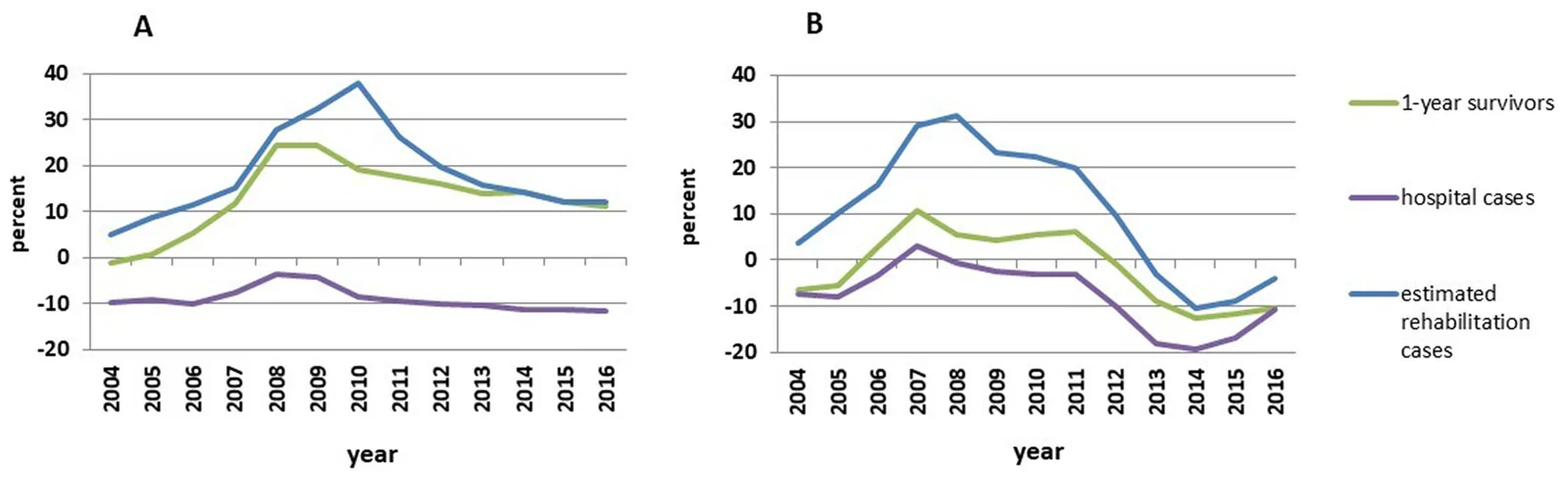
Annual percentage changes since 2003 in the number of incident cases as well as inpatient and rehabilitation cases, 2004–2016. A - Breast cancer (women); B - Prostate cancer (men)

The development of breast cancer incidence since 2003 is characterized by an increase in the number of cases as well as the age-standardized rate through 2008 and a decline thereafter (Fig. 9). The decline in the age-standardized rate from 2008 through 2016 was −14.5%. However, the 2016 incidence rate of 112.2 cases per 100,000 population was still higher than the 2003 rate of 109.3 (Supplement S2). Joinpoint analysis revealed three periods with differing trends (2003–2005, 2005–2008, 2008–2016), with the second period characterized by a significant increase in the number of cases and the third period by a significant decrease (Table 2). Due to fairly constant survival estimates, the trend in the estimated number of 1-year breast cancer survivors is similar to the trend in incident cases.

#### Inpatient care

The number of inpatient cases of breast cancer decreased by approximately 10% from 2003 to 2004. This was followed by a brief increase to a peak in 2008 and a swift decline through 2011. Cases then decreased more slowly through 2016 (Table 2; Fig. 9).

In women, the ratio of inpatient hospital cases to the number of incident cases had a comparatively low value of 1.87 in 2016. From 2003 to 2009, this ratio decreased. In the subsequent period, the numbers varied only slightly (Table 3). For the overall period, the AAPC is −1.6% (95%CI: −1.8 to −1.3%).

#### Rehabilitation

The estimated rehabilitation caseload for women with breast cancer increased by 38% from 2003 to 2010. Since then, this caseload has declined, with particularly sharp drops in 2011 and 2012 (Fig. 9).

The number of rehabilitation cases per 100 1-year breast cancer survivors in 2016 was 49 among women (see Table 1 and Table S2). As can be seen in Fig. 6 and Table 3, this ratio increased until 2010 and decreased to the initial level of 2003. The AAPC for the period 2003 to 2016 is −0.3 (95% CI: −1.0 to 0.4%).

**Fig. 10:**
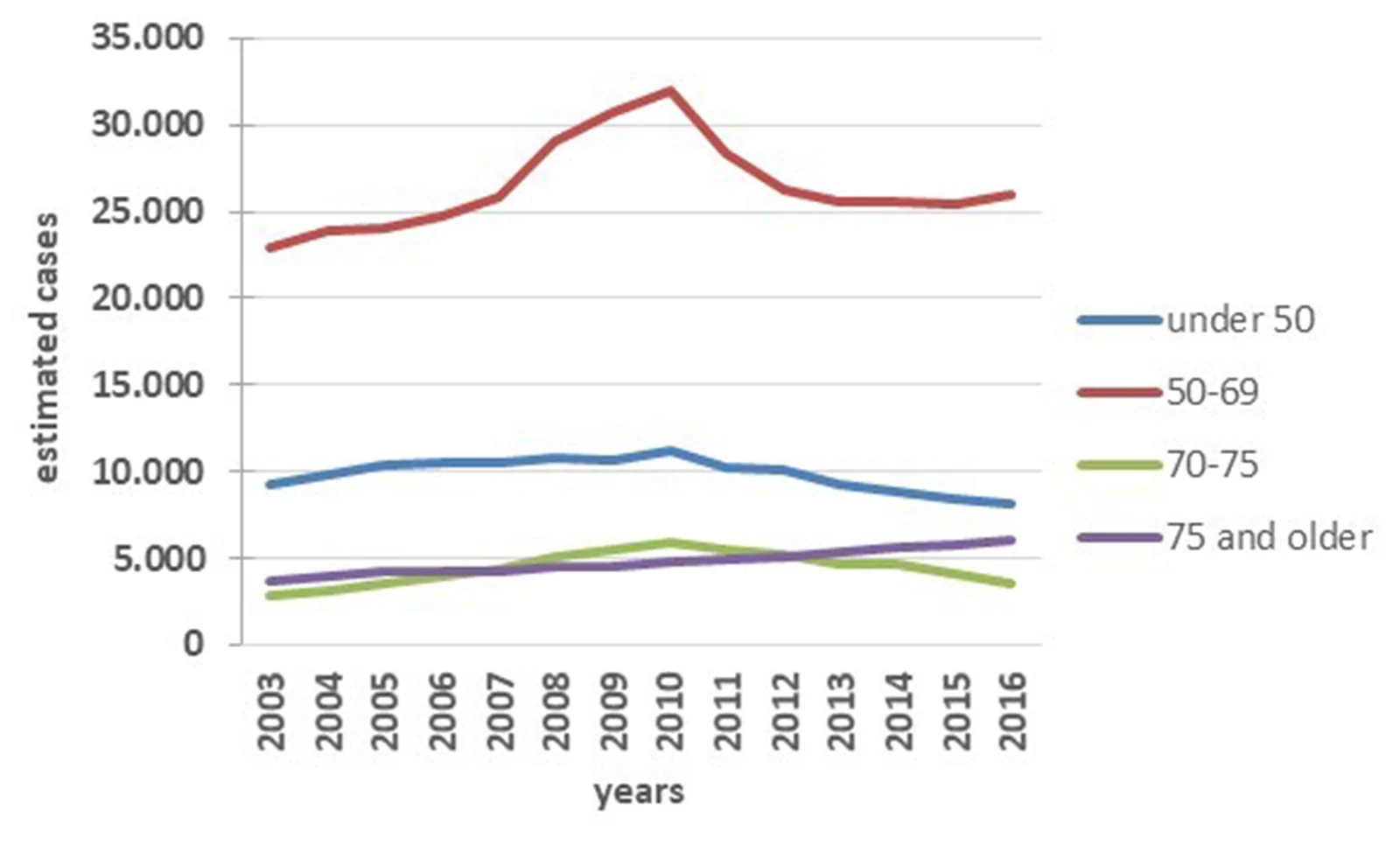
Estimated number of rehabilitation cases 2003–2016 among women with breast cancer (ICD-10 C50), age groups

For breast cancer, different trends can be seen among age groups. As Fig. 10 shows, the aforementioned peak in the number of rehabilitation cases in 2010 is particularly evident in the age groups from 50 to 75 years. In other age groups, this peak is absent or only weakly pronounced.

### Prostate cancer

#### Trends in incidence and 1-year survivors

The trends in the annual number of incident prostate cancer cases, the age-standardized incidence rate and the number of 1-year survivors resemble a hill with two peaks: the highest in 2007 and a lower peak in 2011. The joinpoint regression result shows two periods (2003–2009, 2009–2016), with a significant decrease in the second period (Table 2).

#### Inpatient care

Joinpoint regression detected three periods for inpatient prostate cancer cases in men. In none of these three periods was the trend significant. The ratio between the number of inpatient cases and the number of incident cases of prostate cancer is the lowest of the 4 cancers considered here (1.27). A slight decrease in this ratio can be seen up to around 2014, followed by a sharp increase (see Table 3 and Fig. 5). For the entire period, this results in an AAPC of −0.1% (95% CI: −0.6 to 0.5%).

#### Rehabilitation

The number of rehabilitation cases for men with prostate cancer increased until 2008, only to decline continuously thereafter and fall below the initial level (Table 2; Fig. 9). The relative increase in rehabilitation cases was much more pronounced than the relative increase in 1-year survivors described above.

The number of estimated rehabilitation cases per 100 1-year survivors also initially increased through 2008, then returned to 2003 levels in 2016 (Fig. 6). In the joinpoint analysis, this trend reversal is also evident. An increase is observed until 2008, followed by a decrease (Table 3). Both trends cancel each other out, yielding an AAPC of −0.2% (95%CI: −1.2 to 0.7%).

#### Sensitivity analysis

**Fig. 11:**
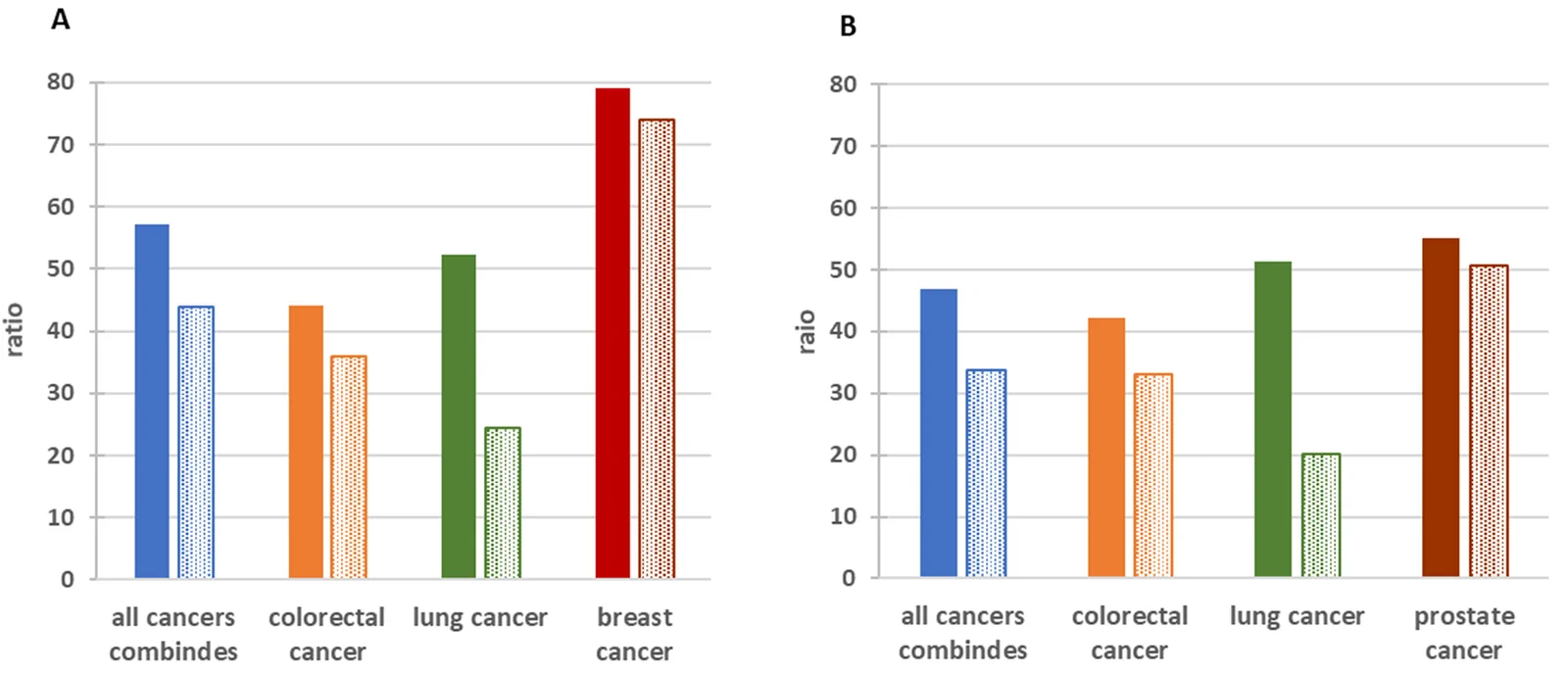
Estimated rehabilitation cases relative to 100 incident cases (dotted bars) vs. estimated rehabilitation cases relative to 100 1-year survivors (fully filled bars), 2016, by type of cancer. A – Women, B – Men

**Fig. 12:**
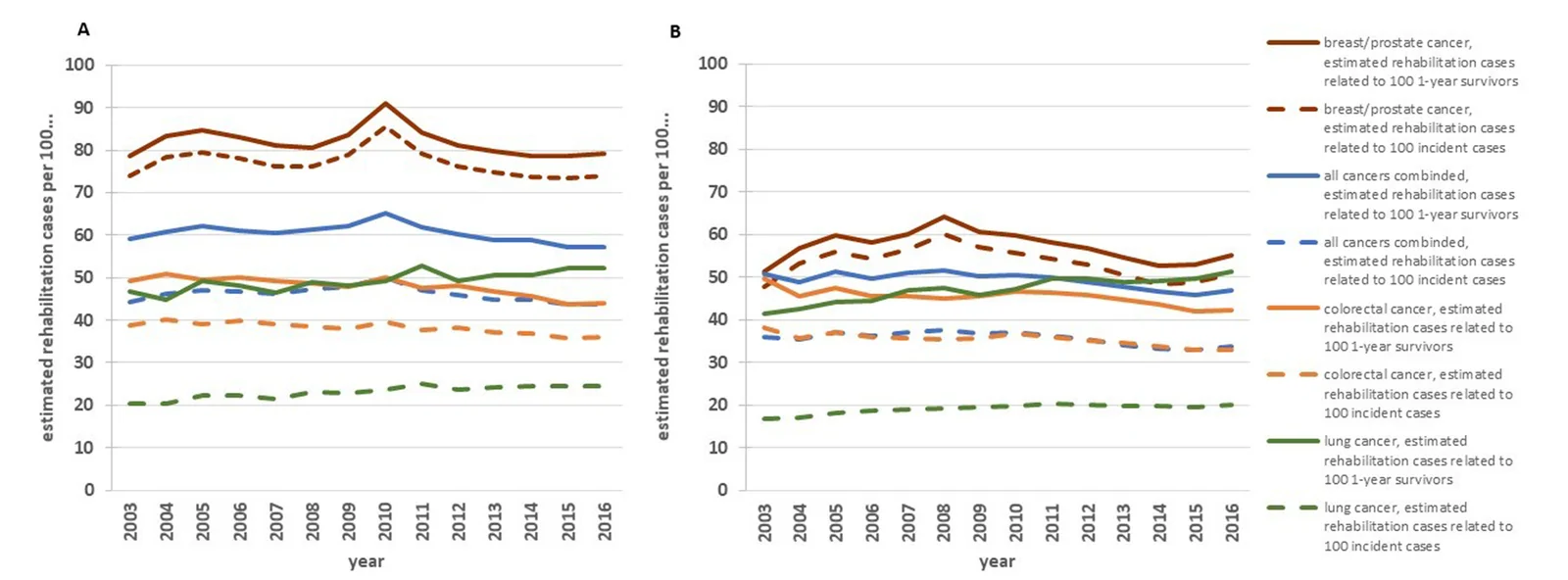
Estimated rehabilitation cases relative to 100 incident cases vs. estimated rehabilitation cases relative to 100 1-year survivors, 2003–2016, by types of cancer. A – Woman, B – Men

As expected, the rehabilitation-to-incidence ratios including all incident cases were lower than the indicator values when including only 1-year survivors. The difference was most pronounced for lung cancer and least pronounced for breast and prostate cancers (Fig. 11). The temporal trends in the indicators appear to be unaffected by the sensitivity analysis (Fig. 12).

There are large differences in lung cancer (for both women and men) between the two rates (see Fig. 11): instead of 52 estimated rehabilitation cases per 100 one-year survivors, there are 25 rehabilitation cases per 100 incident cases. This difference is even greater for men (51 vs. 20). The differences are smaller for colorectal cancer and even smaller for breast cancer (women) and prostate cancer.

## Discussion

### Incidence

Both the annual number of incident cases as well as the crude and age-standardized incidence rates for cancer overall have been declining in recent years, whereas they were rising before the 2010s. The turning point can be located in the 2008–2011 period. Nevertheless, the number of new cases in 2016 was still higher than in 2003 for both women and men.

Demographic ageing, a shift in the age structure of the population towards older age groups, contributes to increasing numbers of cases of age-associated diseases and, consequently, to increasing demand for health care [51–54]. Thus, increasing numbers of cases of age-associated cancers are also to be expected. This is reflected, for example, when the number of cases increases faster or decreases less rapidly than the age-standardized incidence rate. The decline in incident cases since the period 2008–2011 is, therefore, remarkable, since it has taken place despite demographic ageing.

Furthermore, it is likely that cancer screening has influenced the time trends of newly diagnosed cases. Increases in the numbers of screening-detected cancers, particularly breast and prostate cancers, likely contributed substantially to the respective peaks in incidence, whereas colorectal cancer screening has likely prevented the development of invasive tumors [55].

### Inpatient care

In contrast to the estimated number of incident and rehabilitation cases, the number of cases of inpatient cancer care has remained fairly constant over the analysis period, and age-standardized treatment rates have even declined slightly. The former appears surprising at first glance, as one would expect an increase in the number of inpatient cases as a result of demographic ageing alone.

The differences in trends between incidence and hospital cases may be due to shifts in services from the inpatient to the outpatient sector (shift and substitution effects) [56–60]. The following reasons have been discussed for service shifts in oncology care: Newer treatment approaches, particularly in the field of drug therapy, have allowed care to be provided in the home environment since at least the 1990s [9]. The establishment of the Diagnosis Related Groups system in Germany at the beginning of the 2000s exerted strong economic pressure on inpatient care [9]. In addition, there was a gradual expansion of outpatient oncological care: In the mid-1980s, oncological care was still organized entirely on an inpatient basis, but thereafter care was increasingly shifted to the outpatient sector, for example, through the delivery of outpatient care at hospitals or through the development of oncology specialist practices (onkologische Schwerpunktpraxen) or medical care centers (medizinische Versorgungszentren) [61]. In the period between 2003 and 2016, the number of hematology/oncology specialists providing care within the statutory health insurance program rose from 808 to 1,937, more than doubling [62].

For each incident case in a calendar year, there are on average 2.46 (women) or 2.72 (men) inpatient cases. This likely indicates the average extent of multiple inpatient stays per initial cancer diagnosis. Repeated hospitalizations for cancer treatment are common [63], for example due to pre-therapeutic diagnostics, (adjuvant and neoadjuvant) chemo- and/or radiotherapies, complications or further operations (due to recurrence or progression) [43, 64–68]. Very few studies have analyzed the ratio between inpatient treatment and incident cases in Germany. One study used data from a large health insurance company in Saxony-Anhalt, examining patients who died of one of the above-mentioned cancers in 2004. That study included patients with advanced cancer and observed 2.0 hospitalizations per patient with colorectal cancer, 3.3 with lung cancer, 2.5 with breast cancer, and 2.1 with prostate cancer [68]. The data of this study and the results of our analysis are similar. For breast and lung cancer, the values of our study for 2003 are close to the values of the Saxony-Anhalt study (values for 2003/2004). This is somewhat surprising considering the differences in methods. In the Saxony-Anhalt study, only a single federal state was examined, patients were limited to those with advanced cancer, and there were low case numbers. In addition, the Saxony-Anhalt study benefitted from individual-level data. Despite different data and different methodologies, both studies achieved similar results. An additional study of single health insurance enrollees in the state of Hesse published in 2019 reported a median of two hospitalizations per incident case for both women with breast cancer and men with prostate cancer and 3 for men and women with colorectal cancer [63].

Both the number of inpatient cases as well as the ratio of inpatient to incident cases may be driven by epidemiological as well as non-epidemiological factors, the latter including demographic ageing or changes in the organization of healthcare. Our analyses of the relationship between inpatient and incident cases over time as well as the differences in this relationship among the analyzed cancers underscore the difficulties involved in predicting health care needs based solely on epidemiological trends.

### Rehabilitation

For oncological rehabilitation in Germany - as for the entire system of medical rehabilitation - no comprehensive statistic exists. Using a previously developed method [44], we estimated overall use of rehabilitation by cancer patients on the basis of multiple data sources. In addition, we considered 1-year survival among incident cancer cases.

As a result, we found 60 (women) and 48 (men) estimated rehabilitation cases per 100 1-year survivors in Germany in 2016. This means that a considerable number of 1-year survivors apparently do not make use of rehabilitation. Whether this should be taken as evidence of unmet rehabilitative need cannot be definitively inferred from the data sources used here. For example, none of the data sources provides information on subjectively perceived need for rehabilitation. Furthermore, the estimates used here may not capture precise utilization rates. Nevertheless, this indicator can provide information on average use of oncological rehabilitation, allowing a comparison of magnitude and trends among people with different cancers. The use of oncological rehabilitation varies considerably between cancers, from 42% for colorectal cancer (men) to 79% for breast cancer (women) in 2016. The reasons for these differences cannot be determined from the data underlying our analysis.

Oncological rehabilitation participation rates in Germany have been investigated previously with respect to individual types of cancer. For breast cancer, for which most data are available, the participation rates range from 49% to 65% [28–33], for colorectal cancer between 51% [32] and 53% (women) and 44% (men) [35, 37], and for prostate cancer between 45 and 60% [32, 33, 36]. It should be kept in mind that participants in these studies were recruited mostly in clinics [29, 33, 34] or in regional (cancer) registries [28, 31, 32, 35, 36]. This means that their representativeness for Germany as a whole may be limited.

In the case of breast cancer, our estimate of 80% is substantially higher than participation rates reported in other studies. There are a number of potential reasons for these differences. Inclusion and exclusion criteria differed, with some studies excluding women with metastases (e.g. [29]), or persons from certain age groups (e.g. [29, 34]). We excluded women who died within one year of diagnosis from our analyses under the assumption that persons with a poor prognosis would decide against participation in rehabilitation in favor of other types of care. In addition, the length of the follow-up period in the studies could play a role. For example, primary therapy must be completed before the start of rehabilitation [69]. It was unclear whether this criterion was met for some study participants at the time of interview [29, 33, 34]. This could result in a lower utilization of medical rehabilitation in the studies concerned.

Several German [32, 37, 70] and international [5, 71–77] studies have addressed low rehabilitation utilization rates and their causes. Identified causes include factors at the health care level (e.g. lack of information about and motivation for rehabilitation, lack of outpatient services) as well as at the patient level (family, occupational or other private reasons, health status, tumor stage). For the analyses of such access barriers, individual data must be collected, including from non-participants in rehabilitation, as has been done in some international studies (e.g. [5, 71, 75, 77]). However, the special features of the German rehabilitation system and the provision of rehabilitation services almost completely in inpatient facilities make comparison with results from international studies difficult.

The estimated number of oncology rehabilitation cases in Germany peaked in 2010, two years after the peak in the number of incident cases in 2008, as confirmed by the results of the joinpoint analyses. The increase in the number of incident cases and 1-year survivors of breast cancer through 2008 may be an effect of the Mammography Screening Program (MSP) for women, which was rolled out between 2005 and 2009 [40]. In this program, a mammography is offered every two years to women 50 to 69 years old covered by the statutory health insurance, which covers the costs. The MSP aims to detect cancers in early, symptom-free stages and thus improve the chances of successful treatment. Following the rollout, there was a rapid increase in newly diagnosed cases followed by a slower decline in incidence to higher levels than before introduction of the MSP [1, 40]. This pattern typically occurs after introduction of cancer screening, where many prevalent but undiagnosed tumors that would have been detected later are instead detected shortly after introduction. Regarding mammography screening, after this initial peak, incidence has been seen to decline while remaining higher than prior to introduction of screening [78]. In the period 2005 to 2009, 53.8% of eligible women participated in the MSP, approximately 40 thousand cancer cases were detected, and the increase in incidence numbers is attributed to the MSP [79]. The peak in estimated breast cancer rehabilitation cases in 2010 was especially pronounced among women between the ages of 50 and 69 years.

The time lag between epidemiological trends and rehabilitation utilization in breast cancer can probably be attributed to the fact that medical treatment, including long-term therapies with antibodies and (anti-)hormones as well as radiotherapy [66], must be completed before rehabilitation begins [69]. Some forms of therapy may extend over a period of several weeks or months [66]. In addition, it is possible to delay participation in oncological rehabilitation up to two years after cessation of therapy if there is substantial functional impact due to the cancer or due to the consequences of therapy. For these reasons, one may expect a lag in the peak of rehabilitative care for breast cancer compared with the peak in incidence.

### Sensitivity analysis

The differences between the types of cancer regarding the two indicators for rehabilitation are smaller when adjusted for the 1-year survival rate. This means that the differences in the utilization of rehabilitation measures can partly be explained by the one-year survival rate. These reasons may be related to the patient side—such as a lower willingness to participate when prognosis is poor—or to insurance law, for example, because there is little prospect of successful rehabilitation. They are less likely to be due to differences in patients’ expectations regarding the content or quality of rehabilitation.

### Outlook

The SARS-CoV-2 pandemic [80] will most likely further highlight the difficulties in predicting medical and rehabilitation care needs based on epidemiological trends. During the pandemic, there were profound adjustments in the care of people with chronic diseases, including cancer [81–84]. How these will affect the health status of people with cancer, including their rehabilitation needs, will only become apparent in the years to come [85]. In particular, the severity of COVID-19 cases exhibits an age gradient [86], such that epidemiological and demographic factors relevant to cancer incidence and care are also at work here. For future research in this area, the analyses presented here provide methodological tools as well as baseline analyses of the period before the pandemic.

### Strengths and limitations

The analyses presented here have both strengths and limitations.

Regarding the official “hospital diagnosis statistics”, a data check is carried out in the hospitals due to the billing relevance of the diagnoses; additional data checks, such as data checks for item completeness or checks that diagnosis and patient sex are plausible, are carried out by the state statistical offices [45]. However, these data are not subject to comprehensive external quality assurance, for example with regard to validation of diagnoses.

One strength of the study presented is that it provides estimated data on case numbers in oncological rehabilitation as a whole and for various cancers that were previously lacking. This comprehensive view with uniform methods allows a comparison of magnitudes and trends across different cancers and age groups for women and men. Additionally, the data reported here stem from official statistics covering the entire country of Germany. Thus, they are not subject to sample-related errors as can occur with regional or local studies or surveys.

The limitations of this paper stem largely from the different data sources used. Neither the inpatient data nor the rehabilitation data nor the pooled cancer registry data can be linked to other data sets at the level of the individual. However, the use of different datasets is at the same time another strength of the analysis. Despite the lack of individual linkage, our joint analyses of health care and epidemiological data at the aggregate level allow more comprehensive insights than would be possible using only one of the data sources [87]. Another limitation is the lack of information on outpatient cancer care in Germany. Although analyses of outpatient care using statutory insurance claims data may be applied for, standard processing and analysis procedures have not yet been developed. Furthermore, these data only go back to the year 2008, limiting the timeline covered in this paper.

Recently, the Health Data Lab of the Federal Institute for Drugs and Medical Devices has made substantial progress towards providing access to pooled, nationwide claims data for scientific purposes. Furthermore, the Health Data Use Act (Gesundheitsdatennutzungsgesetz) of 2024 [88] paves the way for researchers to be able to link these claims data with cancer registry data. These data, once they become available, will offer an unprecedented opportunity to update and expand on the current analyses.

In summary, our rehabilitation-related analyses provide evidence of discrepancies between cancer incidence and the utilization of oncological rehabilitation. Furthermore, examining the years prior to the rollout of the MSP and considering trends in breast cancer incidence help to explain trends in the case numbers of oncological rehabilitation, whose declining case numbers since 2011 have been reported on previously [32, 89].

## Conclusions

The number of inpatient cancer cases is substantially higher than the number of incident cases, which is likely due to multiple hospital stays in the context of diagnostics and therapy. Trends in the annual numbers of inpatient cases and trends in incidence between 2003 and 2016 differ greatly. Structural changes in the health care system in Germany, such as possible shifts of services from the inpatient to the outpatient care sector, may be partly responsible for these differences.

In oncological rehabilitation, there are about 50 rehabilitation cases per 100 1-year survivors, with differences in this relationship observed according to gender and cancer diagnosis. Whether these results indicate insufficient rehabilitation care cannot be determined based on these data alone. Nonetheless, our nationwide analyses support the findings of previous studies. We observed marked increases and subsequent de-creases in breast cancer incidence and rehabilitation cases among women. These trends coincided with the rollout of the mammography screening program and were most pronounced in the age groups eligible for participation in the program. The peak in rehabilitation cases lagged approximately two years behind the peak in incidence. A corresponding trend in inpatient cases was far less pronounced.

In summary, our analyses show that utilization of inpatient and rehabilitative oncological care is not determined exclusively by epidemiological parameters. Rather, non-epidemiological factors, such as structural changes in the delivery of care or changes in financial incentives, likely play large roles. These caveats also hold for the inverse relationship: it is not possible to infer epidemiological trends based solely on health care utilization data. Future analyses may examine, for example, how projections of healthcare needs can be improved by taking non-epidemiological factors into account.

## Electronic supplementary material

Below is the link to the electronic supplementary material.

Supplementary Material 1

Supplementary Material 2

Supplementary Material 3

Supplementary Material 4

Supplementary Material 5

Supplementary Material 6

Supplementary Material 7

Supplementary Material 8

Supplementary Material 9

## Data Availability

No datasets were generated or analysed during the current study.

## Abbreviations

CCC: Comprehensive Cancer Centers
COVID: Coronavirus Disease
DRG: Diagnosis Related Groups
MSP: Mammography Screening Program
