# Supplementary material for: Cancer care: incidence, hospitalization and medical rehabilitation statistics for Germany from 2003 to 2016
Source: BMC Health Serv Res. 2026 Oct 14;26:1437. doi: 10.1186/s12913-026-15819-x (PMC13682040; doi:10.1186/s12913-026-15819-x)
Supplement: Supplementary file 1 [file 12913_2026_15819_MOESM1_ESM.docx]

**Utilization of inpatient care and medical rehabilitation by oncology patients in relation to cancer incidence in Germany 2003 to 2016**

Enno Nowossadeck and Benjamin Barnes

**Additional files**

Additional file S1: Table: Incident cases and case numbers in inpatient and rehabilitative care, by sex and cancer site, 2016.

Additional file S2: Table: Indicators of inpatient and rehabilitative care, by sex and cancer site, 2016.

Additional file S3: Table: Estimation of rehabilitation cases relative to 1-year survivors, all cancer combined, 2016.

Additional file S4: Table: Incidence: cases, crude rates and age-standardized rates, 2003-2016

Additional file S5: Joinpoint Analyses: Ratio of the number of inpatient cases to the number of incident cases, men

A – C18-C20

B – C33-C34

C – C61

Additional file S6: Figure: Joinpoint Analyses: Ratio of the number of inpatient cases to the number of incident cases, women

A – C18-C20

B – C33-C34

C – C61

Additional file S7: Figure: Joinpoint Analyses: Ratio of estimated number of rehabilitation cases per 100 1-year survivors, men

A – C18-C20

B – C33-C34

C – C61

Additional file S8: Figure: Joinpoint Analyses: Ratio of estimated number of rehabilitation cases per 100 1-year survivors, women

A – C18-C20

B – C33-C34

C – C61
